# Supplementary material for: Iron status influences mitochondrial disease progression in Complex I-deficient mice
Source: eLife. 2023 Feb 17;12:e75825. doi: 10.7554/eLife.75825 (PMC10030112; doi:10.7554/eLife.75825)

Figure 3 – Source Data 1 – Olfactory Bulb

GFAP

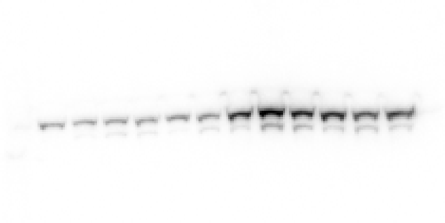

GFAP

Total Protein

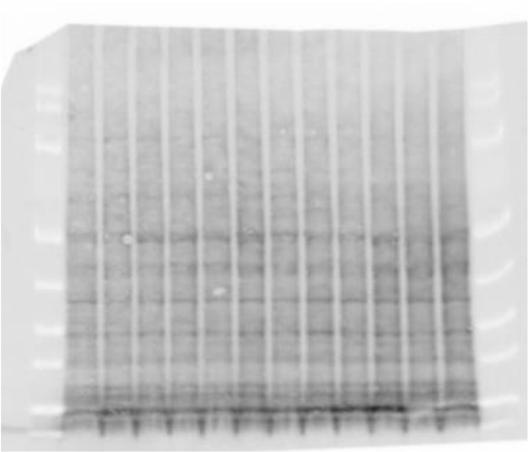

Figure 3 – Source Data 1 – Cerebellum

GFAP

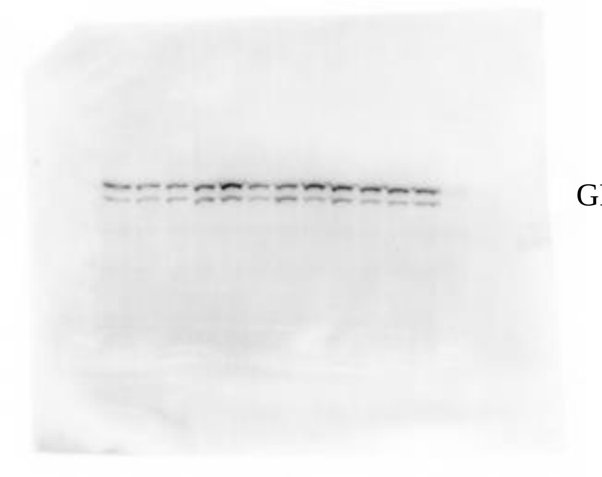

GFAP

Total Protein

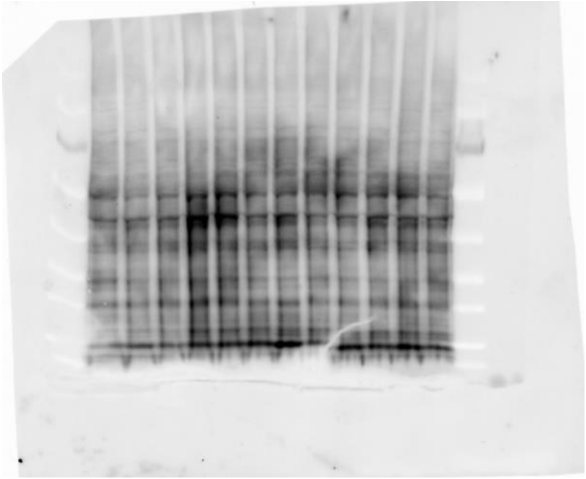

Figure 3 – Source Data 1 – Cortex

GFAP

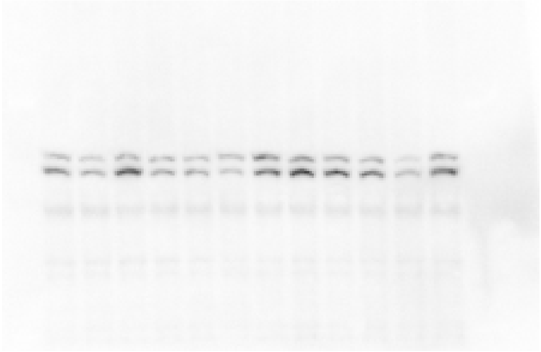

GFAP

Total Protein

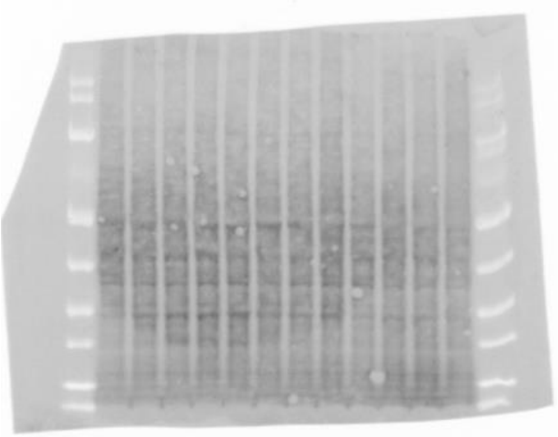

Supplement: Figure 3—source data 1. [file elife-75825-fig3-data1.zip › Figure 3 - Source Data 1/Figure 3 - Source Data 1.pdf]
