# Supplementary figures and images for: Iron status influences mitochondrial disease progression in Complex I-deficient mice
Source: eLife. 2023 Feb 17;12:e75825. doi: 10.7554/eLife.75825 (PMC10030112; doi:10.7554/eLife.75825)

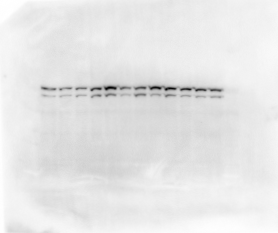

Supplement: Figure 3—source data 1. [file elife-75825-fig3-data1.zip › Figure 3 - Source Data 1/Figure 3 - Source Data 1 Cerebellum GFAP.tif]

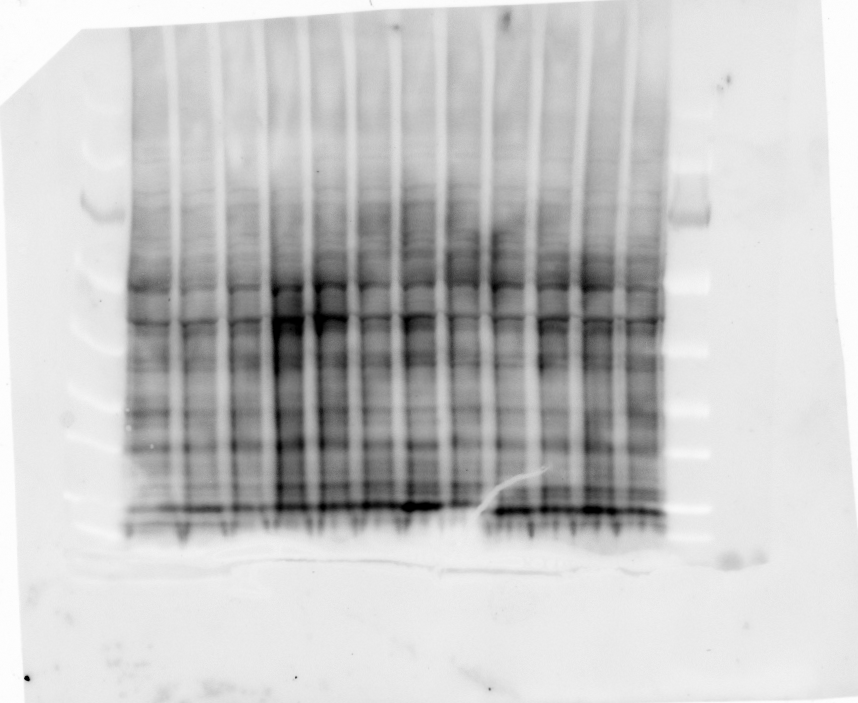

Supplement: Figure 3—source data 1. [file elife-75825-fig3-data1.zip › Figure 3 - Source Data 1/Figure 3 - Source Data 1 Cerebellum Total.tif]

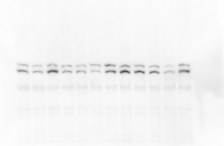

Supplement: Figure 3—source data 1. [file elife-75825-fig3-data1.zip › Figure 3 - Source Data 1/Figure 3 - Source Data 1 Cortex GFAP.tif]

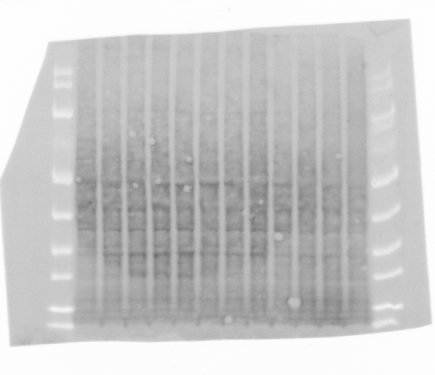

Supplement: Figure 3—source data 1. [file elife-75825-fig3-data1.zip › Figure 3 - Source Data 1/Figure 3 - Source Data 1 Cortext Total.tif]

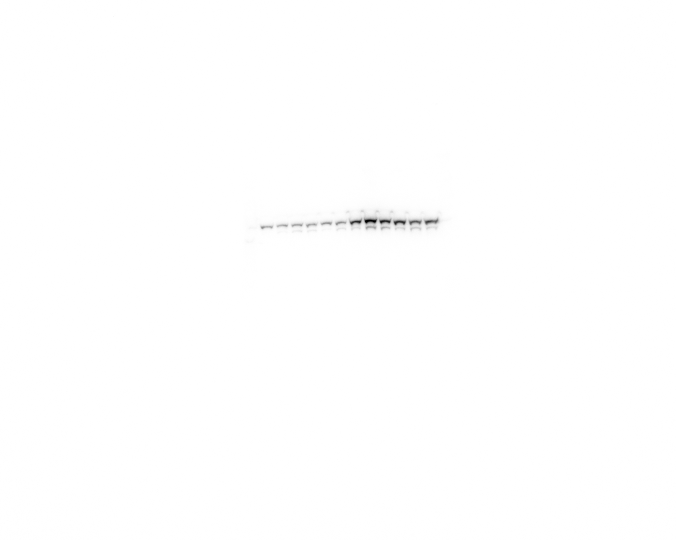

Supplement: Figure 3—source data 1. [file elife-75825-fig3-data1.zip › Figure 3 - Source Data 1/Figure 3 - Source Data 1 OlfBulb GFAP.tif]

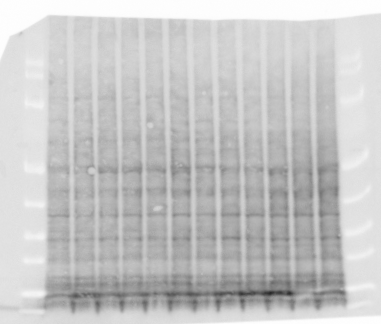

Supplement: Figure 3—source data 1. [file elife-75825-fig3-data1.zip › Figure 3 - Source Data 1/Figure 3 - Source Data 1 OlfBulb Total.tif]

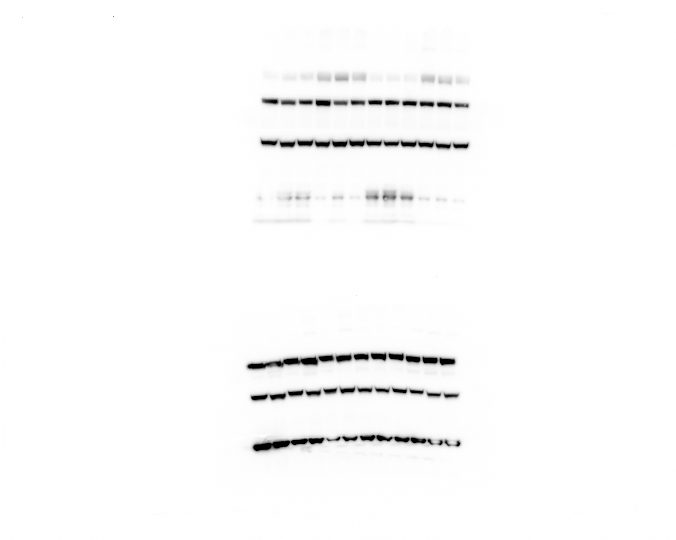

Supplement: Figure 4—source data 1. [file elife-75825-fig4-data1.zip › Figure 4 - Source Data 1/Figure 4 - Source Data 1 Actin M.tif]

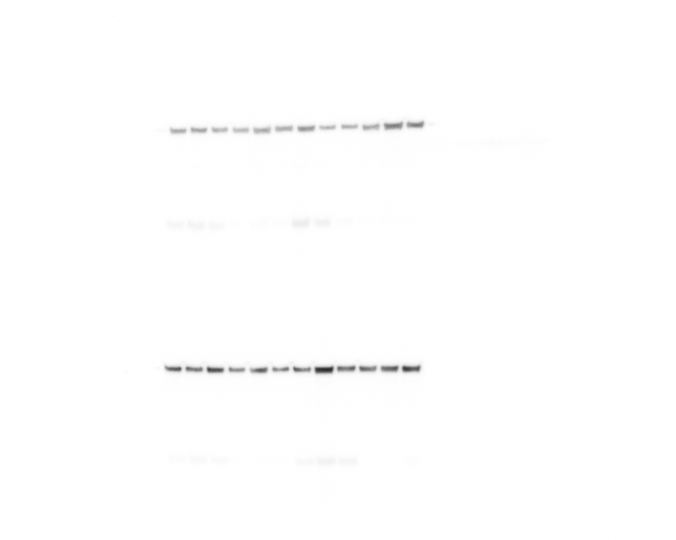

Supplement: Figure 4—source data 1. [file elife-75825-fig4-data1.zip › Figure 4 - Source Data 1/Figure 4 - Source Data 1 DMT1 M.tif]

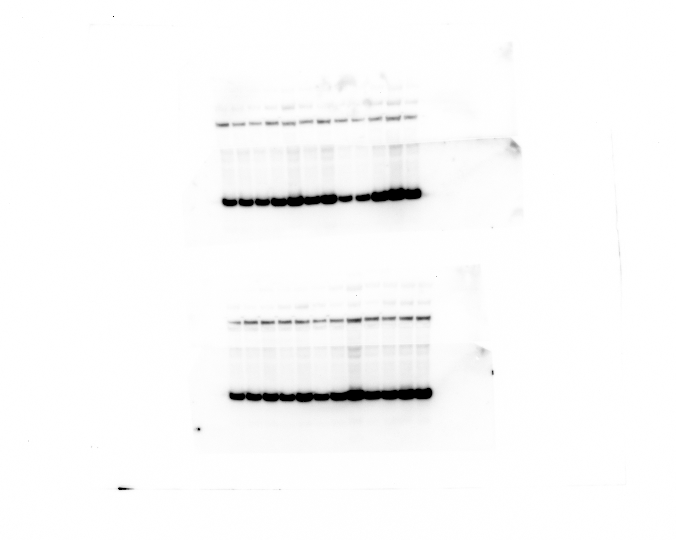

Supplement: Figure 4—source data 1. [file elife-75825-fig4-data1.zip › Figure 4 - Source Data 1/Figure 4 - Source Data 1 FPN1 M.tif]

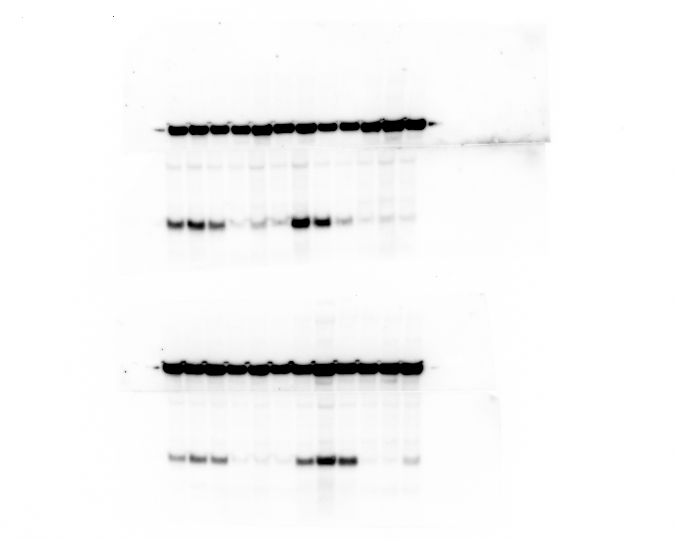

Supplement: Figure 4—source data 1. [file elife-75825-fig4-data1.zip › Figure 4 - Source Data 1/Figure 4 - Source Data 1 FTH1 M.tif]

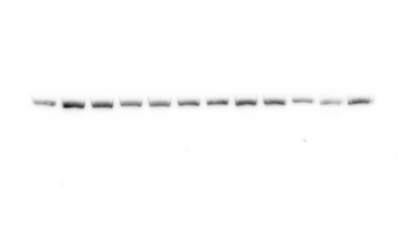

Supplement: Figure 4—source data 1. [file elife-75825-fig4-data1.zip › Figure 4 - Source Data 1/Figure 4 - Source Data 1 IRP1 F.tif]

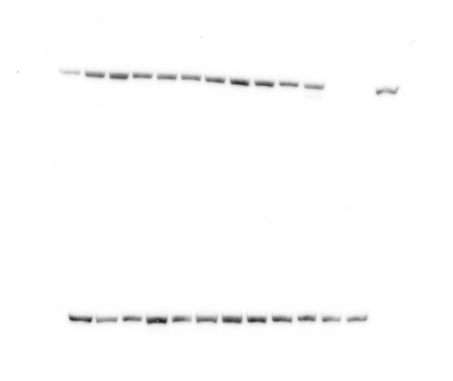

Supplement: Figure 4—source data 1. [file elife-75825-fig4-data1.zip › Figure 4 - Source Data 1/Figure 4 - Source Data 1 IRP1 M.tif]

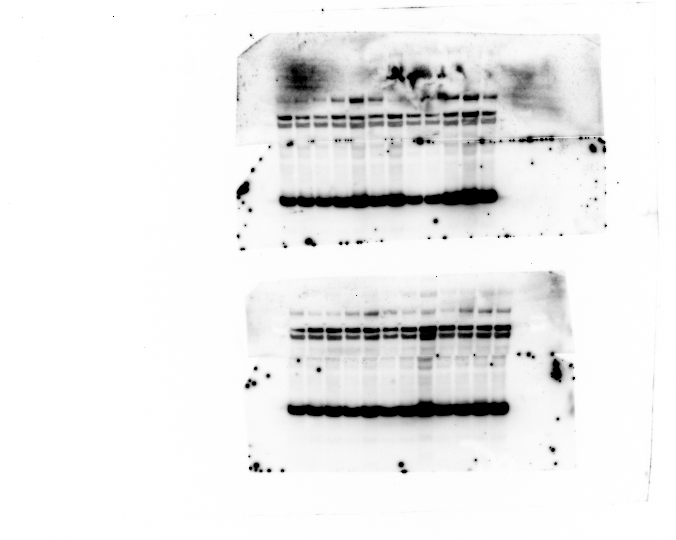

Supplement: Figure 4—source data 1. [file elife-75825-fig4-data1.zip › Figure 4 - Source Data 1/Figure 4 - Source Data 1 IRP2 F.tif]

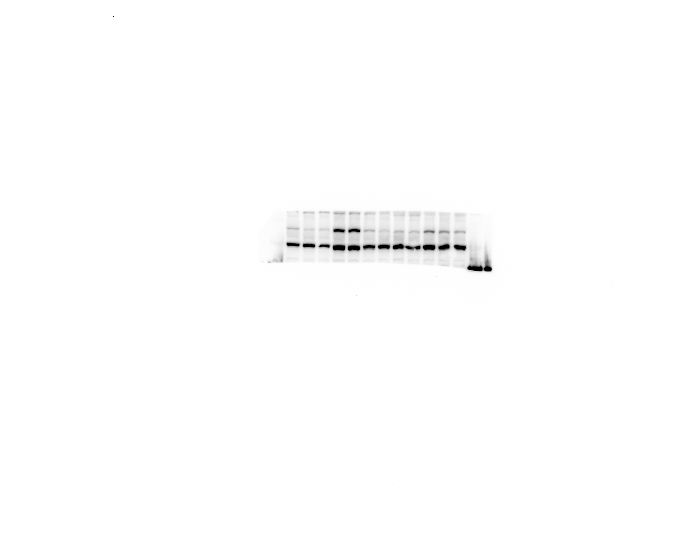

Supplement: Figure 4—source data 1. [file elife-75825-fig4-data1.zip › Figure 4 - Source Data 1/Figure 4 - Source Data 1 IRP2 M.tif]

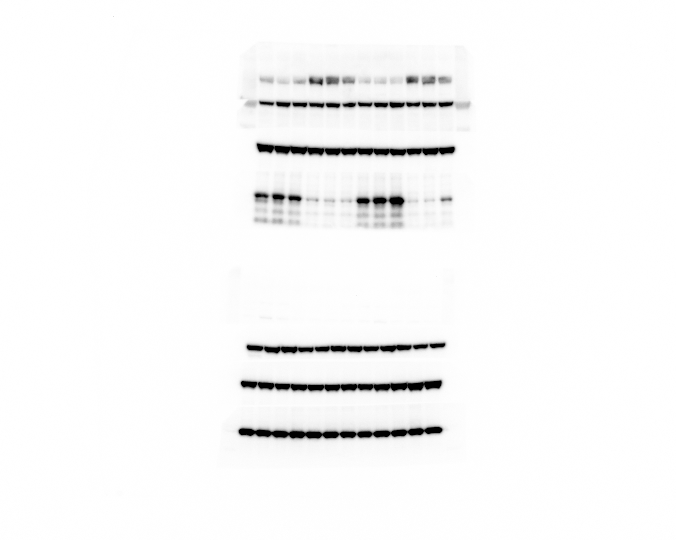

Supplement: Figure 4—source data 1. [file elife-75825-fig4-data1.zip › Figure 4 - Source Data 1/Figure 4 - Source Data 1 TFR1 FPN1 FTH1 DMT1 F.tif]

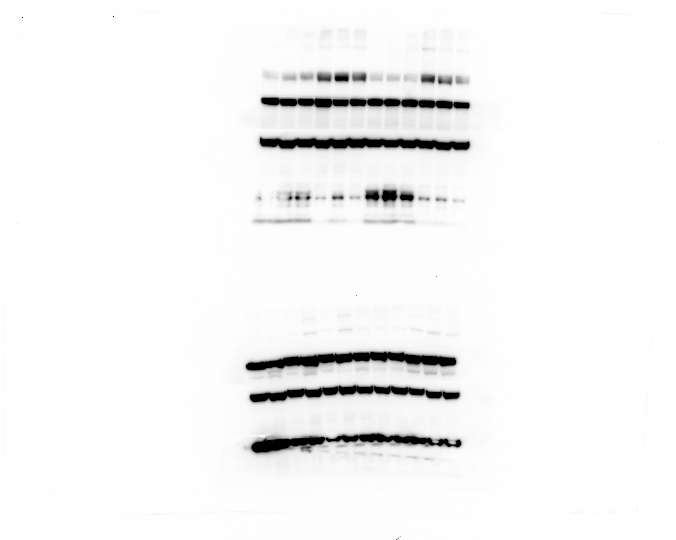

Supplement: Figure 4—source data 1. [file elife-75825-fig4-data1.zip › Figure 4 - Source Data 1/Figure 4 - Source Data 1 TFR1 M.tif]

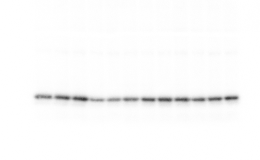

Supplement: Figure 4—figure supplement 2—source data 1. [file elife-75825-fig4-figsupp2-data1.zip › Figure 4 - figure supplement 2 - Source Data 1/Figure 4 - figure supplement 2 - Source Data 1 A.tif]

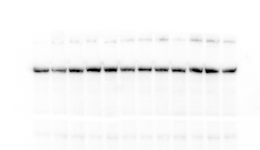

Supplement: Figure 4—figure supplement 2—source data 1. [file elife-75825-fig4-figsupp2-data1.zip › Figure 4 - figure supplement 2 - Source Data 1/Figure 4 - figure supplement 2 - Source Data 1 B.tif]

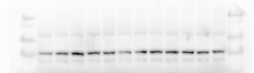

Supplement: Figure 4—figure supplement 2—source data 1. [file elife-75825-fig4-figsupp2-data1.zip › Figure 4 - figure supplement 2 - Source Data 1/Figure 4 - figure supplement 2 - Source Data 1 C.tif]

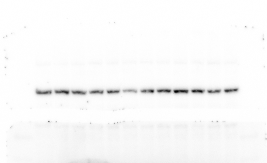

Supplement: Figure 4—figure supplement 2—source data 1. [file elife-75825-fig4-figsupp2-data1.zip › Figure 4 - figure supplement 2 - Source Data 1/Figure 4 - figure supplement 2 - Source Data 1 D.tif]

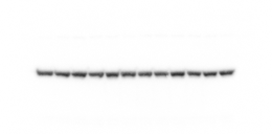

Supplement: Figure 4—figure supplement 2—source data 1. [file elife-75825-fig4-figsupp2-data1.zip › Figure 4 - figure supplement 2 - Source Data 1/Figure 4 - figure supplement 2 - Source Data 1 E.tif]

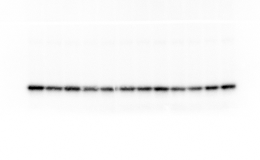

Supplement: Figure 4—figure supplement 2—source data 1. [file elife-75825-fig4-figsupp2-data1.zip › Figure 4 - figure supplement 2 - Source Data 1/Figure 4 - figure supplement 2 - Source Data 1 F.tif]

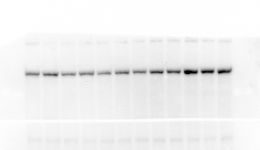

Supplement: Figure 4—figure supplement 2—source data 1. [file elife-75825-fig4-figsupp2-data1.zip › Figure 4 - figure supplement 2 - Source Data 1/Figure 4 - figure supplement 2 - Source Data 1 G.tif]

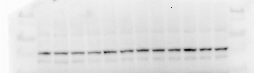

Supplement: Figure 4—figure supplement 2—source data 1. [file elife-75825-fig4-figsupp2-data1.zip › Figure 4 - figure supplement 2 - Source Data 1/Figure 4 - figure supplement 2 - Source Data 1 H.tif]

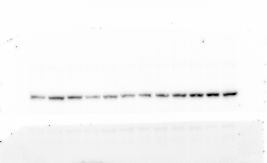

Supplement: Figure 4—figure supplement 2—source data 1. [file elife-75825-fig4-figsupp2-data1.zip › Figure 4 - figure supplement 2 - Source Data 1/Figure 4 - figure supplement 2 - Source Data 1 I.tif]

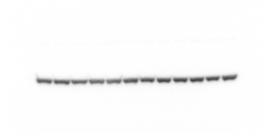

Supplement: Figure 4—figure supplement 2—source data 1. [file elife-75825-fig4-figsupp2-data1.zip › Figure 4 - figure supplement 2 - Source Data 1/Figure 4 - figure supplement 2 - Source Data 1 J.tif]
