# Supplementary material for: Iron status influences mitochondrial disease progression in Complex I-deficient mice
Source: eLife. 2023 Feb 17;12:e75825. doi: 10.7554/eLife.75825 (PMC10030112; doi:10.7554/eLife.75825)
Supplement: Figure 4—source data 1. [file elife-75825-fig4-data1.zip › Figure 4 - Source Data 1/Figure 4 - Source Data 1.pdf]

<u>Figure 4 – Source Data 1 – Female</u>

High exposure (TFR1, FPN1, FTH1, DMT1)

Gel 1

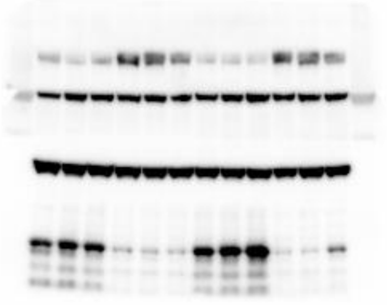

TFR1
FPN1
actin
FTH1

Gel 2

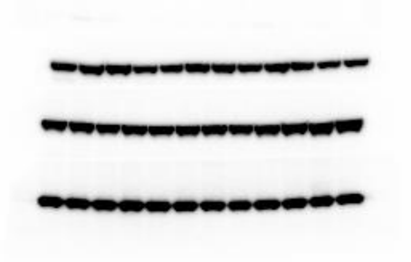

DMT1
actin
SOD2

Low Exposure (actin)

Gel 1

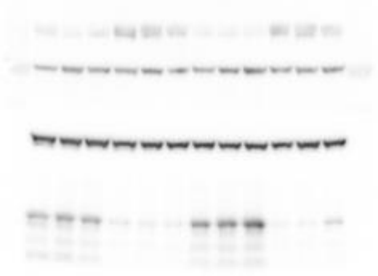

TFR1
FPN1
actin
FTH1

Gel 2

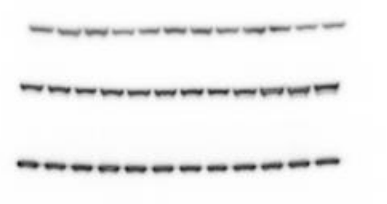

DMT1
actin
SOD2

IRP1

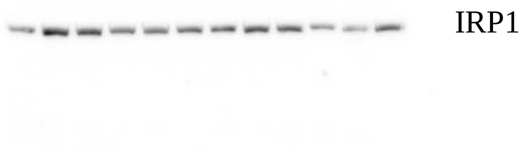

IRP2

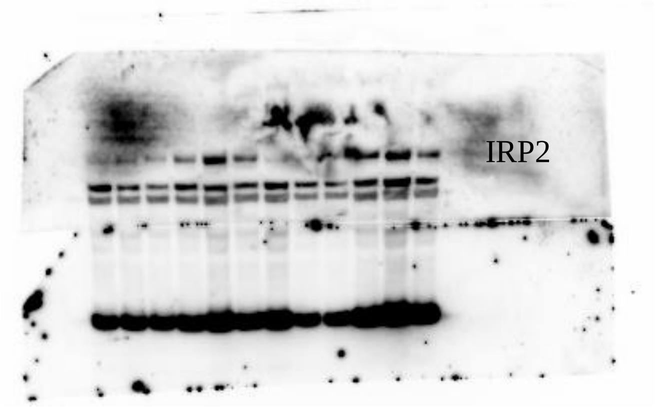

<u>Figure 4 – Source Data 1 – Male</u>

FTH1

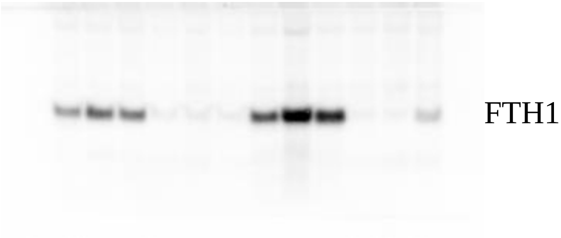

FTH1

DMT1

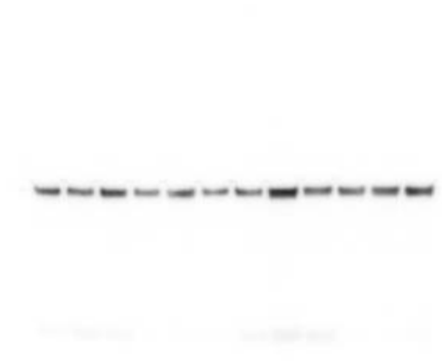

DMT1

TFR1

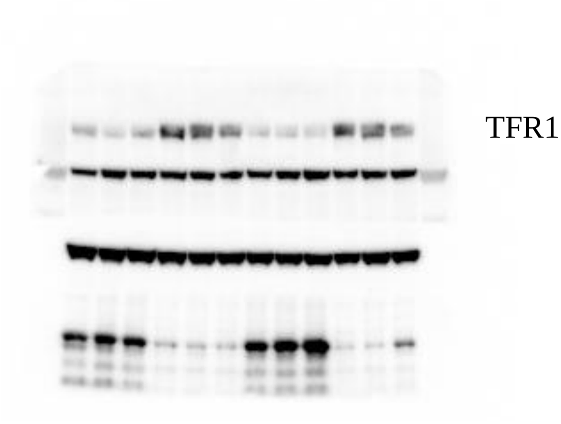

TFR1

FPN1

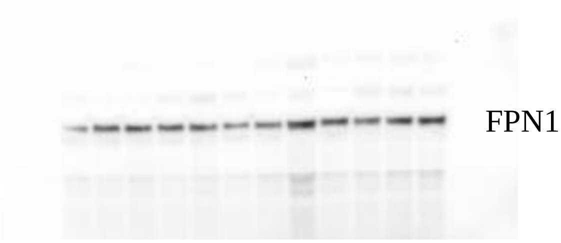

FPN1

IRP1

IRP1

IRP2

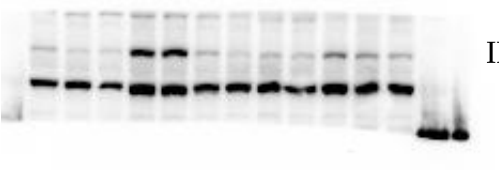

IRP2

Actin

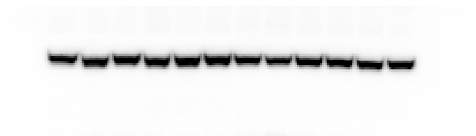

actin
