# Supplementary material for: Iron status influences mitochondrial disease progression in Complex I-deficient mice
Source: eLife. 2023 Feb 17;12:e75825. doi: 10.7554/eLife.75825 (PMC10030112; doi:10.7554/eLife.75825)
Supplement: Figure 4—figure supplement 2—source data 1. [file elife-75825-fig4-figsupp2-data1.zip › Figure 4 - figure supplement 2 - Source Data 1/Figure 4-figure supplement 2 - Source Data 1.pdf]

**<u>Figure 4-figure supplement 2 – Source Data 1 – Female (Brain)</u>**

FTH1

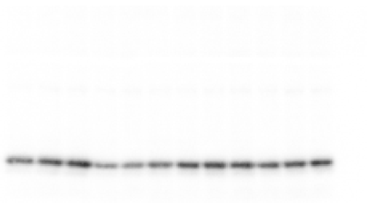

FTH1

TFR1

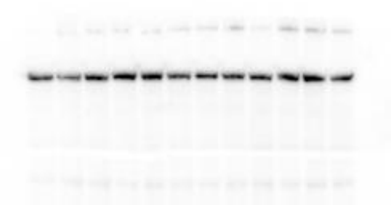

TFR1

FPN1



FPN1

DMT1

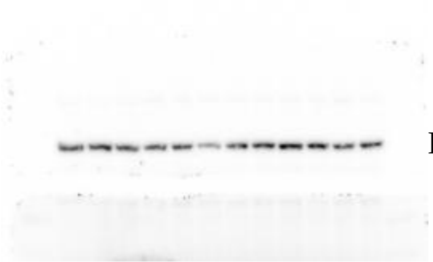

DMT1

Actin

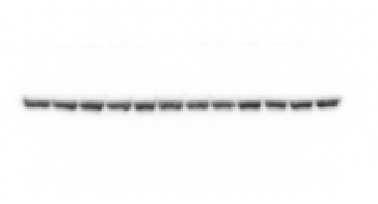

actin

**Figure 4-figure supplement 2 – Source Data 1 – Male (Brain)**

FTH1

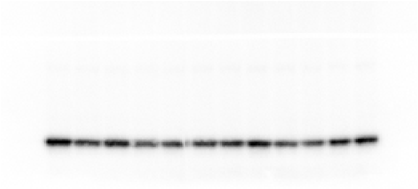

FTH1

TFR1

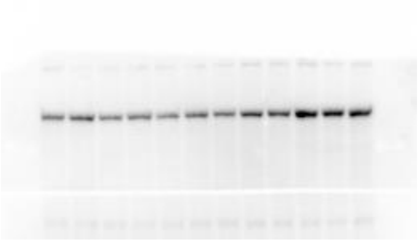

TFR1

FPN1

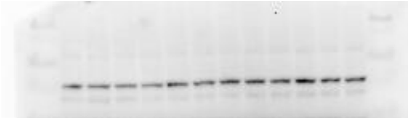

FPN1

DMT1

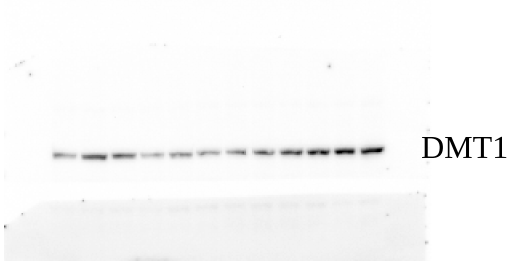

DMT1

Actin

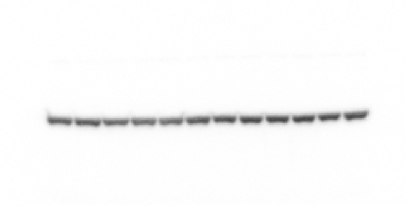

actin
